# Dental Hygiene

**Published:** 1864-05

**Authors:** W. W. Allport


### DENTAL HYGIENE,

Or how to Save the Teeth from Decay. A Paper read before the Chicago Dental Society, by W. W. Allport.

THAT many diseases can be prevented by proper hygienic regulations as easily as they can be cured by medical treatment, is a principle well established in medical science. The common axiom of medicine, that the knowledge of what a disease is, constitutes half its cure, is not more true than that in order to know how to prevent a disease, we must know its cause. This knowledge no man can possess by intuition; it can be ascertained only by observation and reason. It is from facts observed, and statistics gathered, by medical men, that they have been convinced that certain diseases are produced by certain causes. We are taught by statistics that the mortality is far greater to a given number of inhabitants, and that the mean duration of life is much shorter in populous towns, than in rural districts, of the same climate. This shows that a city residence is much less conducive to health, than one in the country. We also know that, in the crowded and filthy wards of a city, certain types of disease are much more apt to prevail, and that individuals attacked by these diseases are much less likely to recover than those who reside in the cleanly wards with large yards surrounding their houses, where they are supplied with an abundanco of pure air. Hence we are drawn to the irresistible conclusion that cleanliness and pure air are important requisites to good health, and that filth and impure air are prolific and exciting causes of disease.

From observations often repeated, and statistics carefully collected, it has been ascertained that diseases may be engendered, not only by crowding too many persons into dark and badly ventilated rooms, but by the accumulation of filth and dirt. Whole columns of figures might be given to demonstrate the correctness of this position; but for our purpose it is hardly necessary. We will content ourselves with a single illustration.

It is well known by medical men, that at certain seasons, typhus fever is especially rife in densely populated and filthy parts of populous towns. Statistics show that in those parts of London where the population is one to every one hundred and eighty square yards, the proportion of deaths from typhus to the whole mortality is 131 out of 2,289, or about six per cent. of the whole; where the population is one to thirty-five square yards, it is 349 out of 3,428, or over ten per cent., nearly double the ratio in the less crowded district.

It has frequently happened that places once considered unhealthy have become proverbial for the health of their inhabitants, after a proper system of sewerage and drainage has been introduced. This is especially the case with our own city of Chicago. So fully have facts of this kind been established that the proverb, "An ounce of prevention is worth a pound of cure," has become a favorite maxim all over the world. Hence the willingness of all large cities to submit to almost any amount of taxation for the prevention of disease or the removal of its cause.

These principles apply with as much force in dental as in medical science, and they have just as important a relation to the welfare of the people in the one case as in the other. It is as true of the decay of the teeth as of the many forms of disease which attack the more vital organs and demand the more immediate attention of the medical practitioner, that it is due to well known causes—and that in this case, as in the other, to prevent the disease, we must remove its cause. Clearly as it seems to be demonstrated that disease is engendered by impure air and filth, we doubt whether there could be as many facts brought forward to substantiate this opinion, as can be adduced to prove that the premature decay and loss of so many thousands of teeth, is induced by well known chemical action, arising from the accumulation of filth between and around the teeth, which the patient himself can prevent, and which no one else can. Surely, the proverb, "An ounce of prevention is worth a pound of cure," is no more true in medicine than it is in dentistry.

And yet the above facts and principles, while they are appreciated and acted upon in the one case, are practically ignored and set aside in the other, by a large majority of people. Why should there be this difference? Is it because people consider sound teeth of no consequence? Is it because they care nothing for the inconvenience and pain caused by the decay and loss of the teeth? Is it because they are unwilling to be at any expense to secure a healthy condition of the teeth, while they readily pay thousands to preserve their general health? The very fact that they do cheerfully pay large sums to the dentist when they become aware of the necessity of employing him, is of itself sufficient to give a decided negative to all such questions.

The truth is, that people do not fully understand the importance of *preventive* treatment in regard to the teeth; and this is because our profession has not taken the pains to enlighten and instruct them on the subject as the medical profession has done in respect to the prevention of other diseases. We have something to learn in this respect from our brother practitioners of the older branch of medicine, and if we do our duty we have here a great work. Hitherto the dental profession has spent its energies chiefly in treating diseases of the teeth, and in reaching the highest point of professional skill and dexterity. We have been content to take the patient with his teeth already diseased, sadly dilapidated, or hopelessly ruined, and to restore them to health by filling or otherwise treating them, or else remove them with as little pain as possible, and to supply their loss by the best artificial substitutes. This done, he has been sent away, deeply impressed with the idea that he has been peculiarly fortunate in securing the highest skill in the profession. No pains have been spared in putting in hard filling and polishing them till they equal the enamel itself in hardness and finish. His teeth have been cleansed till they rival pearls in whateness, and with mirror in hand he is invited to see how beautifully the work has been done. The dentist pockets his fee. and the patient departs as ignorant of the cause of the decay as when he came. But with this the work of a really good dentist does not end, and more than merely curing a sick man constitutes the whole duty of the true physician. Our desire and effort should be not only to do our work well, to restore diseased teeth to a healthy condition, or to provide the best artificial substitute (which at best must come far short of good natural teeth), but so to instruct our patients as to the causes of decay, and so to impress upon them the necessity of absolute cleanliness of the teeth, that the necessity of dental operations on themselves or their children may be less frequent.

To cure disease scientifically, is a high attainment of the healing art, and wins for the medical profession the well-deserved meed of public praise. To forestall disease, and thus obviate the necessity of cure, is still nobler, and when by hygienic and sanitary regulations on a large scale, a whole community is saved from the ravages of disease and pestilence, it entitles that profession to the highest rewards of philanthropy. It is this higher ground of *prevention* that the dental profession is now called upon to occupy. Admirable as is surpassing professional skill, it is not enough, to perform successfully difficult operations, to excel in mechanical execution, and to be able to insert a better set of artificial teeth than our next door neighbor. If we would elevate our calling to its true dignity as a public benefactor, we must above all things, by proper instruction, seek to prevent the necessity of frequent dental operations.

What should we think of a physician who should treat successfully a large nnmber of cases of typhus or intermittent fever, and yet, though the cause was well known and could be easily removed, should neglect to tell the neighborhood what it was, and to impress upon them the necessity of its removal. We might admire the knowledge and skill which could effect the cure, but it would be at the expense of his reputation for philanthropy. And what should we think of the medical faculty as a body, if, when they knew that a certain cause was producing a fatal epidemic and hurrying thousands to their graves, they should go on treating the cases and pocketing the fees, without making any effort to spread a knowledge of the proper precautionary measures.

Such, in a measure, is the position of the dental profession. Knowing full well the causes of the decay of the teeth, and that three-fourths of all that are lost could have been saved by being kept clean, we have been content to treat them, when diseased, giving merely a little *oral* instruction to each patient (and quite too little of that even), leaving the public at large in total ignorance as to the importance of keeping the teeth perfectly clean.

What now is our duty in this matter, not only as professional men, but as philanthropists! I answer—not only to give such oral instruction to those with whom we come immediately in contact as patients, but to disseminate through popular journals and other publications, information on this subject which will arrest and fix popular attention, and convince all of the desirableness and necessity of proper care and absolute cleanliness of their teeth, in order to prevent their decay.

